# Research on Gating Fusion Algorithm for Power Grid Survey Data Based on Enhanced Mamba Spatial Neighborhood Relationship

**DOI:** 10.3390/s24216980

**Published:** 2024-10-30

**Authors:** Aiyuan Zhang, Jinguo Lv, Yu Geng, Xiaolei Wang, Xianhu Li

**Affiliations:** 1 School of Geomatics and Urban Spatial Informatics, Beijing University of Civil Engineering and Architecture, Beijing 100044, China; 2108570023107@stu.bucea.edu.cn (A.Z.); 2108570023087@stu.bucea.edu.cn (Y.G.); 15138513773@163.com (X.W.); 2 Shanghai Aerospace Space Technology Co., Ltd., Shanghai 201306, China; lxh@sastspace.com

**Keywords:** selective state space model, spatial–spectral fusion, spatial proximity constraint, deep learning, power grid surveying, gated fusion

## Abstract

In power grid surveying, it is often necessary to fuse panchromatic and multispectral imagery for the design of power lines. Despite the abundance of deep learning networks for fusing these images, the results often suffer from spectral information loss or structural blurring. This study introduces a fusion model specifically tailored for power grid surveying that significantly enhances the representation of spatial–spectral features in remote sensing images. The model comprises three main modules: a TransforRS-Mamba module that integrates the sequence processing capabilities of the Mamba model with the attention mechanism of the Transformer to effectively merge spatial and spectral features; an improved spatial proximity-aware attention mechanism (SPPAM) that utilizes a spatial constraint matrix to greatly enhance the recognition of complex object relationships; and an optimized spatial proximity-constrained gated fusion module (SPCGF) that integrates spatial proximity constraints with residual connections to boost the recognition accuracy of key object features. To validate the effectiveness of the proposed method, extensive comparative and ablation experiments were conducted on GF-2 satellite images and the QuickBird (QB) dataset. Both qualitative and quantitative analyses indicate that our method outperforms 11 existing methods in terms of fusion effectiveness, particularly in reducing spectral distortion and spatial detail loss. However, the model’s generalization performance across different data sources and environmental conditions has yet to be evaluated. Future research will explore the integration of various satellite datasets and assess the model’s performance in diverse environmental contexts.

## 1. Introduction

With the advancement of remote sensing technology, methods of obtaining imagery have become increasingly diverse, and remote sensing images are widely applied across various industries. In the field of power grid surveying, two types of remote sensing images are commonly used: multispectral images (MSIs) with low spatial resolution, but high spectral resolution, and panchromatic (PAN) images with high spatial resolution, but low spectral resolution. Since a single sensor or satellite cannot capture remote sensing images with both high spatial and high spectral resolution, researchers often employ image fusion techniques to combine the complementary characteristics of MSIs and PAN images, ultimately achieving images with high resolution in both spatial and spectral domains [1].

The purpose of this study is to develop a novel image fusion technology that provides power grid survey designers with more comprehensive and clear information about the surrounding objects, terrain, and real environment of the survey area. We aim to generate images with high spatial and spectral resolution by fusing high-resolution MSIs and PAN images, thereby assisting survey designers in accurately identifying houses, vegetation distribution, and ground obstacles that may affect power grid construction without the need for on-site inspections. Furthermore, by enhancing the recognition of key geographical features, our fused images help optimize route planning and avoid crossing sensitive areas, such as residential areas, rivers, and forests.

Existing fusion techniques for MSIs and PAN images mainly originate from the idea of pan-sharpening [2], which can be divided into four categories [3]: component substitution (CS), multiresolution analysis (MRA) [2], variational optimization (VO), and model-based methods [4].

The core principle of CS lies in separating spatial structure from the MSI and then replacing the separated spatial information components with high spatial resolution images to generate a new image. Methods such as intensity–hue–saturation transformation [5], principal component analysis [6], and Gram–Schmidt (GS) transformation [7] are relatively fast in computation, but often result in spectral distortion.

MRA applies decomposition methods to extract spatial details from PAN images and then inject them into the MSI to obtain the fusion result. Examples include discrete wavelet transform [8], contourlet transform [9], Laplacian [10], and generalized Laplacian pyramid [11]. However, the fusion quality of these methods is overly dependent on the accuracy of spatial detail extraction.

VO-based methods usually convert the fusion into an inverse problem and use models, such as the Bayesian paradigm [12], total variation regularization [13], and sparse representation models [14], to represent the relationships between MSIs, PAN images, and the fused image. But choosing the appropriate regularization term depends on prior knowledge of the image.

In recent years, inspired by a series of breakthroughs in deep learning in various computer vision applications, model-based methods have been widely studied. Compared with traditional methods, deep learning-based methods construct complex network structures to learn the mapping relationship between HS and PAN images, achieving a better balance between spatial and spectral quality. Pan-sharpening neural network (PNN) [15] is a pioneering work that introduces domain-specific knowledge into network design to achieve spectral preservation. PanNet [16] and its extensions involve adding the upsampled MSI to the output for lossless spectral information propagation, but this introduces redundant information, leading to spectral distortion. To better preserve spatial and spectral information, researchers have adopted dual-branch structures to distinguish between spatial and spectral information extraction. GANs, with their powerful generation capabilities and adversarial training mechanisms, can well maintain the consistency of spectral and spatial information. For example, PSGAN [17] directly takes PAN images and MSIs as inputs to the generator to obtain the fusion result, while the discriminator is only used for adversarial training. MDSSC-GAN [18], AN-GAN [19], PanColorGAN [20], FGF-GAN [21], and other models are successful cases based on GANs that make the generated image consistent with the original high-resolution image in both space and spectrum. In addition, researchers have proposed fusion networks based on diffusion models, such as DCDMF [22], SpectralDiff [23], and DKDMN [24]. These methods use the spatial relationships between pixels in the image to improve the fusion effect. However, diffusion models require high computational resources and time, bringing limitations to practical applications. The Mamba model has linear time complexity for sequence modeling, which can well solve the above bottleneck problems. RSM was the first to transfer the Mamba model to the remote sensing field, proposing OSSM to extract large spatial scale and multi-directional spatial features from ultra-high-resolution remote sensing images.

Although many methods have been specifically designed for the task of fusing panchromatic and hyperspectral images, generating high spatial and spectral quality fused HS images remains challenging due to long-range dependencies, difficulty in capturing spatial relationships between objects, and loss of spatial–spectral details in the power grid field.

To address these challenges, this paper proposes a multi-scale progressive gated fusion model based on Mamba and attention mechanisms. The main contributions of this model are summarized as follows.

To endow the Mamba model with the ability to process three-dimensional remote sensing image data, this paper innovatively proposes a spatial–spectral embedding layer. This layer cleverly achieves effective integration of spatial and spectral features in two dimensions, embedding the complex three-dimensional information of remote sensing images into the model.

To fully extract global and local features of remote sensing images, this study proposes the TransforRS-Mamba model. This model combines the sequence processing capabilities of the Mamba model with the attention mechanism of the Transformer architecture, introducing a spatial proximity perceives attention mechanism (SPPAM), enhancing the ability to capture complex object proximity relationships.

This study innovatively proposes a spatial proximity constraint gating fusion module (SPCRGF), which introduces a spatial constraint matrix during the fusion process to accurately capture and emphasize key object features such as houses, rivers, and vegetation, enhancing the practical application of the fusion results in power grid surveying.

The rest of this paper is organized as follows. Section 2 introduces related work, and Section 3 details the proposed TransforRS-Mamba and fusion strategy. We present experimental results in Section 4 and report conclusions in Section 5.

## 2. Related Work

### 2.1. State Space Model

State Space Model (SSM) are a fundamental concept in signal processing and systems theory, wherein the core idea is to transform a one-dimensional input sequence x(t)∈R into a multidimensional latent state h(t)∈R^N^ to generate an output y(t)∈R. Mathematically, SSM are often represented as linear ordinary differential equations (ODEs):

(1) $$\begin{array}{l} h^{\prime }\left(t\right)=Ah\left(t\right)+Bx\left(t\right)\\ y\left(t\right)=Ch\left(t\right)+Dx\left(t\right) \end{array}$$

The behavior of a system in an SSM is defined by a set of parameters, which include the state transition matrix A∈R^N×N^, the projection parameters B,C∈R^N×1^, and the direct feedthrough connection D∈R.

In practice, to transition from continuous parameters A and B to discrete parameters $\bar{A}$ and $\bar{B}$, the zero-order hold (ZOH) principle is applied by introducing a time-scale parameter Δ. The discretization of the equations can be expressed as follows:

(2) $$\begin{array}{l} \bar{A}=\exp\left(\Delta A\right)\\ \bar{B}={\left(\Delta A\right)}^{-1}\left(\exp\left(\Delta A\right)-I\right)\cdot \Delta B\\ h_{t}=\bar{A}h_{t-1}+{Bx}_{t}\\ y_{t}=Ch_{t} \end{array}$$

### 2.2. Mamba and Variants

The Mamba model is a pioneering work in the field of deep learning, focusing on the development of efficient methods for sequence modeling. It has its roots in State Space Model (SSM), which were traditionally used in control theory and signal processing, with the Kalman filter being a notable example. The S4 model, an early iteration of an SSM, was designed to capture long-term dependencies within sequences through a structured approach. The S6 model advanced this concept by incorporating a selective mechanism, which improved not only the model’s efficiency, but also its ability to dynamically adjust to the relevance of input data.

The Mamba model builds upon the S6 model’s foundation, enhancing sequence modeling capabilities to linear time complexity through the use of efficient selective scanning operations and algorithms optimized for hardware. This advancement has spurred research into integrating Mamba’s linear-time modeling with other existing models, aiming to achieve a balance between performance and computational efficiency. Over the past year, scholars have developed a variety of Mamba-based visual models for diverse tasks, such as classification, detection, segmentation, and medical imaging.

In the realm of multimodal generation tasks, VL-Mamba [25] marks the initial attempt to leverage Mamba to mitigate the computational expenses associated with the Transformer architecture. Another notable model, CMViM [26], concentrates on the application of multimodal representation learning to 3D high-resolution medical imaging. Its primary innovation is the introduction of contrastive learning methods that address inconsistencies in representations across different modalities.

Although the Mamba model shows promise in the processing of natural images, its application in remote sensing imagery faces certain challenges. The characteristics of remote sensing imagery, which include high spatial resolution, rich spectral information, and wide coverage areas, place higher demands on the model. Panchromatic and multispectral images in remote sensing often have long-range dependencies and spectral–spatial details, requiring the Mamba model to capture and understand spatial features and contextual information over large areas. Therefore, these are important difficulties that must be addressed when migrating the Mamba model to the field of remote sensing. Our research delves into the potential of Mamba, aiming to offer novel approaches for tasks such as remote sensing image fusion.

## 3. Method

**Figure 1:**
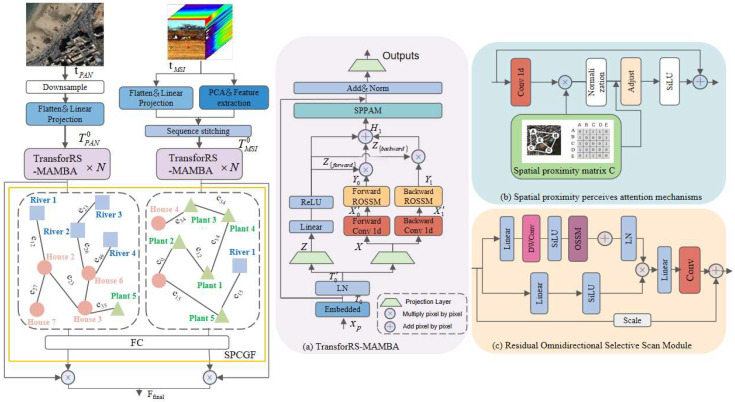
The fusion network in our study commences with a spectral–spatial embedding layer for the preliminary processing of the input imagery, succeeded by several TransforRS-MAMBA modules. These modules are engineered to extract and augment features through their transformative capabilities. The network culminates with a spatial proximity constraint gated fusion module, which amalgamates the derived spectral–spatial features, ensuring that the fused output preserves superior spatial and spectral acuity. (**a**) Depicts the TransforRS-MAMBA module; (**b**) illustrates the spatial proximity perception attention mechanism; and (**c**) represents the residual panoramic selective scanning module.

Figure 1 illustrates our network architecture, which is divided into three key stages: spectral–spatial embedding, feature extraction, and feature fusion. We start by downsampling the PAN image in relation to the original MSI, with the downsampling ratio set as the spatial resolution ratio between the two, resulting in images t_PAN_∈R^H×W×C^ and t_MSI_∈R^H×W×C^. Subsequently, these images are decomposed and embedded across spatial and spectral dimensions to form a feature tensor T_0_∈R^B×M×D^ that incorporates spectral–spatial data.

The feature tensor T_0_ is then subjected to feature extraction via a series of RS-Mamba blocks, yielding a sequence of features H_l_∈R^B×M×D^, each denoted by l∈{1, 2, …, l} and rich with contextual information. These features are further refined by the Spatial Pyramid Pooling Attention Module (SPPAM) to produce feature sequences F_PAN_ and F_MSI_, which capture local spatial details. Ultimately, the network employs a spatial neighborhood constraint gated fusion module to produce an image X that achieves high resolution in both spatial and spectral dimensions.

### 3.1. Spectral–Spatial Embedding Layer

**Figure 2:**
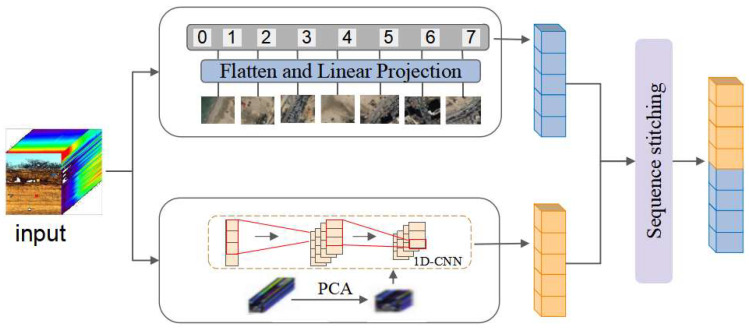
Spectral–spatial embedding layer.

The conventional Mamba model is tailored for optical images with one-dimensional spectral features. In contrast, hyperspectral images offer both spectral and spatial two-dimensional characteristics. This paper introduces a specialized spectral–spatial embedding layer to address the distinct attributes of hyperspectral imagery; this is shown in Figure 2.

First, the 2-D image t∈R^H×W×C^ is transformed into flat 2-D patches $x_{p}\in R^{J\times \left(P^{2}\cdot C\right)}$, where H and W represent the dimensions of the input image, C is the number of channels, and P is the size of the image patches. J denotes the number of patches. Subsequently, the patches x_p_ are linearly project onto vectors of size D, and positional embeddings E_pos_∈R^J+1×D^ are added, as follows:

(3) $$T_{\mathrm{space}}=\left[x_{cls};x_{p}^{1}W;x_{p}^{2}W;\ldots ;x_{p}^{J}W\right]+E_{pos}$$

where $T_{\mathrm{space}}\in R^{B\times M\times D}$ denotes a sequence of tokens, B represents the batch size, M signifies the length of the sequence, and D indicates the dimension of the State Space Model (SSM). $W\in R^{\left(p^{2}\cdot C\right)\times D}$ is a learnable projection matrix, which is a trainable matrix used to transform image patches into a format that the model can process effectively.

Simultaneously, Principal Component Analysis (PCA) is conducted on the spectral dimension for dimensionality reduction, selecting the optimal spectral bands; the results are then fed into a 1D-Convolutional Neural Network (1D-CNN) for the extraction of spectral features.

(4) $$T_{\mathrm{spectrum}}=U^{T}\cdot diag\left(\sum \right)\cdot V$$

where $T_{\mathrm{spectrum}}\in R^{B\times M\times D}$, U represents the matrix of left singular vectors obtained from PCA, and diag(Σ) is a diagonal matrix containing the singular values from the PCA decomposition, sorted in descending order to reflect the relative importance of each principal component. V is the matrix of right singular vectors, with its columns representing the projection coefficients of the data along the principal component directions.

Finally, the sequence tensor is concatenated to obtain the final tensor sequence T_0_∈R^B×M×D^ that encapsulates both spatial and spectral information.

### 3.2. TransforRS-Mamba

Drawing on the strengths of hybrid architectures in language modeling, we introduce TransforRS-Mamba, an innovative feature extraction module. It integrates the Mamba model’s ability to efficiently capture long-term dependencies with the Transformer’s proficiency in local feature recognition. Additionally, the inclusion of a spatial proximity matrix further strengthens the spatial connectivity of features. This approach allows for a comprehensive feature extraction that balances both global and local insights within the remote sensing data.

#### 3.2.1. RS-Mamba

The Mamba block is a sequence-to-sequence module with equal input and output dimensions. It begins by taking an input T_0_ and normalizing it to obtain ${T\prime }_{0}$. Then, it expands the dimensions through two input linear projections to vectors X∈R^B×M×E^ and Z∈R^B×M×E^ of dimension E, where E is the extended state dimension. For X, the algorithm processes it from both forward and backward directions. For each direction, a one-dimensional convolution Conv1d is applied to X to obtain X’_0_.

**Figure 3:**
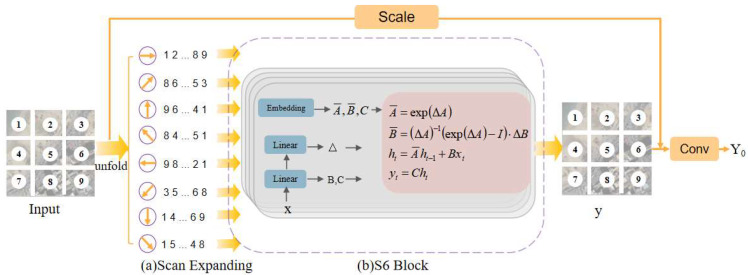
Overview of proposed ROSSM module, which consists of two interconnected steps: (**a**) scan expanding and (**b**) S6.

The Residual Omnidirectional Selective Scan Module (ROSSM) is tailored for remote sensing images, which can exhibit spatial features in any orientation due to their acquisition from a satellite’s perspective. This is shown in Figure 3. The module performs a full-directional selective scan across horizontal, vertical, and both diagonal axes, creating eight sets of image patch sequences. Subsequently, the obtained sequence is input into the S6 module. X’_0_ is then linearly transformed into A_0_, B_0_, C_0_, and ∆_0_.

(5) $$\begin{array}{l} {\bar{A}}_{0}=\Delta_{0}⊗Parameter_{0}^{A}\\ {\bar{B}}_{0}=\Delta_{0}⊗B_{0}\\ {\bar{C}}_{0}=C_{0} \end{array}$$

The module uses Δ_0_ to discretely transform A_0_, B_0_ into ${\bar{A}}_{0},{\bar{B}}_{0}\in R^{B\times M\times E\times N}$. Here, Δ is processed through a softplus operation to ensure it remains positive, as it will be used for computing temporal scale transformations.

After this transformation, a State Space Model is applied to compute the final output y. However, the process of generating a 1D sequence from a 2D feature map through selective scanning results in some features that are spatially adjacent in 2D being distant from each other in the 1D marked sequence. This increased distance may lead to the loss of local feature details. To address this, a separate local convolution is introduced by the ROSSM to assist in the restoration of neighborhood similarity. Finally, another adjustable scaling factor s’∈R^C^ is used in a residual connection to obtain the final output Y_0_ of the ROSSM.

For Z, it sequentially passes through a linear layer and the SiLU activation function for nonlinear transformation, resulting in the gating parameters $Z^{\prime }$, Z_{forward}_ and Z_{backward}_. These parameters are then used to perform gating operations on Y_0_ in their respective directions.

(6) $$\begin{array}{l} Z_{\left\{forward\right\}}=f_{\theta_{i}}\left(Z\right)\\ Z_{\left\{backward\right\}}=f_{\varpi_{i}}\left(Z\right) \end{array}\;\begin{array}{l} {Y^{\prime }}_{\left\{forward\right\}}=Z_{\left\{forward\right\}}\cdot Y_{0}\\ {Y^{\prime }}_{\left\{backward\right\}}=Z_{\left\{backward\right\}}\cdot Y_{1} \end{array}$$

where ${Y^{\prime }}_{\left\{forward\right\}}\in R^{B\times M\times E}$ and ${Y^{\prime }}_{\left\{backward\right\}}\in R^{B\times M\times E}$ represent the vectors after gating operations, and · signifies element-wise multiplication. Essentially, the gating mechanism acts as a means of information filtering. By combining Z_{forward}_ and Z_{backward}_ with the activation function, the model can control which information is retained or emphasized and which is suppressed or diminished during the forward and backward processing.

Finally, features are enhanced by adding $Z^{\prime }$, ${Y^{\prime }}_{\left\{forward\right\}}$, and ${Y^{\prime }}_{\left\{backward\right\}}$ pixel-wise to obtain the output token sequence H_l_∈R^B×M×D^.

#### 3.2.2. Spatial Proximity Perceives Attention Mechanism

**Figure 4:**
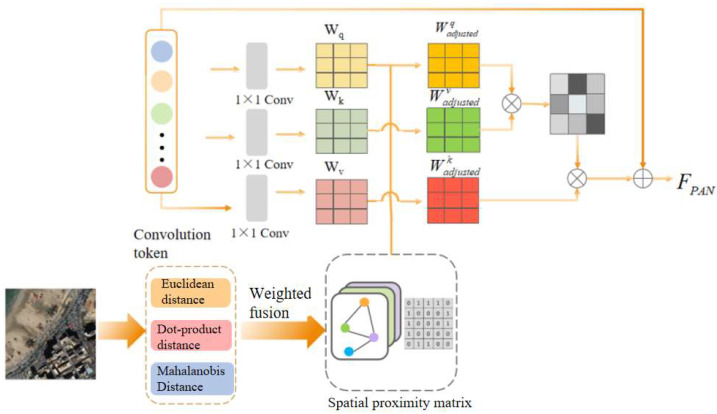
Spatial proximity perceives attention mechanism (SPPAM).

In the context of power grid surveying, the spatial relationships between objects are crucial, as they provide the precise relative positioning, size, and distribution of objects. However, the H_l_ obtained from the RS-Mamba block exists in the form of a one-dimensional sequence, which overlooks the spatial structural information it retains. To address this issue, this study designed the SPPAM, which integrates spatial proximity constraints and uses residual connections to compensate for the spatial relationships between features. This is shown in Figure 4.

Each pixel of the original image is treated as a graph node, with the pixel’s original features serving as node features. Three types of spatial distance metrics are used to calculate the spatial relationships between each pair of pixel nodes: Euclidean distance (Ed) [27], Dot-product distance (Dd) [28], and Mahalanobis Distance (Md) [29]. These metrics result in three different proximity matrices C_x ij_, where C_x ij_ represents the spatial proximity between pixel i and pixel j, quantifying the spatial similarity between pixels. Then, by using the entropy weighting method, these matrices are weighted and fused to form a comprehensive spatial proximity matrix C, which automatically determines the importance of each distance metric.

First, tokens are input into the self-attention mechanism, allowing the model to consider not only the features of each token itself, but also the relationships between tokens within the sequence. Subsequently, to enable the model to consider the spatial relationships between pixels when processing features, the spatial proximity matrix C is used to adjust the features after convolution. The aim is to introduce spatial proximity information to adjust the self-attention weights W_adjusted_, so that the model considers not only the sequence positions of tokens, but also their spatial relationships when calculating attention weights. This allows the model to utilize both spatial and spectral information when extracting features, leading to a more comprehensive understanding and analysis of image data, thereby improving the accuracy and efficiency of image processing. Finally, through residual connections and normalization layers, the results are combined with spatial attention to obtain feature sequences F_PAN_ and F_MSI_ containing rich local spatial proximity information.

(7) $$\begin{array}{l} e_{i}=-\frac{1}{\ln\left(n\right)}\sum_{j=1}^{n}p_{ij}\ln\left(p_{ij}\right)\\ w_{i}=\frac{1-e_{i}}{\sum_{j=1}^{n}\left(1-e_{j}\right)}\\ C=w_{Ed}\cdot C_{Ed}+w_{Dd}\cdot C_{Dd}+w_{Md}\cdot C_{Md}\\ W_{adjusted}^{Q}=W_{Q}+\alpha \cdot C\cdot V\\ W_{adjusted}^{K}=W_{K}+\beta \cdot C\cdot V\\ W_{adjusted}^{V}=W_{V}+\gamma \cdot C\cdot V \end{array}$$

The entropy value e_i_ of matrix C_ij_ is calculated using the following formula, where p_ij_ is the normalized value of the element in the i-th row and j-th column of matrix C_i_, and n is the number of rows or columns in the matrix. C_Ed_, C_Dd_, and C_Md_ correspond to the spatial proximity matrices for Euclidean distance, Dot-product distance, and Mahalanobis Distance, respectively. W_i_ is the entropy weight of each matrix, and α, β, and γ are coefficients used to adjust the weights.

### 3.3. Spatial Proximity Constraint Gating Fusion Module

To ensure that the fusion process accurately captures the characteristics of objects surrounding the power lines, this study proposes the Spatial Proximity Constraint Gating Fusion Module (SPCGF), as shown in Figure 1. Under the guidance of spatial constraints between different features, adjacent feature information is progressively aggregated, focusing the fusion results on the impact of features such as buildings, rivers, and vegetation on line design and mitigating risks.

First, each pixel v in the feature maps F_PAN_ and F_MSI_ is considered as a node i in the graph, forming a node set V. Edges e_ij_ between nodes are constructed based on their spatial proximity, forming an edge set E.

Next, based on the spatial distance between nodes i and j, a Gaussian function is used to attenuate W_ij_ and assign weights w_ij_ to each edge e_ij_ in the graph.

(8) $$W_{ij}=e^{-\frac{d_{ij}^{2}}{2\sigma^{2}}}$$

These weights reflect the spatial proximity between nodes i and j. Then, a graph convolutional network is employed to iteratively learn the complex spatial relationships between nodes and update the feature representations of the nodes. The graph convolution operation can be represented as:

(9) $$h_{i}^{l+1}=\sigma \left(\sum_{j\in Ν\left(i\right)}W^{\left(l\right)}h_{j}^{\left(l\right)}+b^{\left(l\right)}\right)$$

where h_i_^(l)^ represents the feature representation of node i at layer l, N(i) is the set of neighboring nodes of node i, W^(l)^ is the weight matrix at layer l, b^(l)^ is the bias term, and σ is a nonlinear activation function.

Ultimately, the output from the graph convolutional network is used to transform the feature representations of any two nodes h_i_ and h_j_ into a spatial proximity constraint matrix S through cosine similarity $S_{ij}=\frac{h_{i}h_{j}}{\left\|h_{i}\right\|\left\|h_{j}\right\|}$. The matrix S is then input into a fully connected layer to compute the gating weights G for each pixel location.

The computed gating weights G are utilized to control the fusion of panchromatic features F_PAN_ and multispectral features F_MSI_. This process ensures that the fusion is guided by the spatial relationships between features, enhancing the integration of information from both types of imagery.

(10) $$F_{Final}=G⊗F_{PAN}+\left(1-G\right)⊗F_{MSI}$$

Constructing a spatial proximity constraint matrix facilitates the precise capture of geospatial features, enhancing the recognition capabilities for terrain, landforms, and key geographic objects. The gating mechanism dynamically determines the contribution of different features during the fusion process, allowing for a more adaptive and context-aware integration of information.

## 4. Experiments and Analysis

### 4.1. Model Environment

In this study, we selected a Python 3.9 environment for model development and training. The experimental setup is based on an AMD Ryzen 7 5800H processor (AMD, Santa Clara, CA, USA) and an NVIDIA GeForce RTX 4090 GPU (NVIDIA, Santa Clara, CA, USA). The training data are batched with an input block of 32, while the testing and validation data are batched with an input block of 16. A total of 200 epochs are iterated over. The training process is expected to last 150 h, with an initial learning rate set at 0.01. An SGD optimizer is used, configured with a learning rate of 2 × 10^−5^, momentum of 0.9, and weight decay of 0.0001. All experiments are completed on a Linux operating system.

### 4.2. Dataset

In this study, we employed the QuickBird (QB) [30] dataset and the GF-2 satellite dataset [31] for our experiments. The QB satellite provides Multi-Spectral Imagery (MSI) and Panchromatic (PAN) images with spatial resolutions of 2.44 m and 0.61 m, respectively. The images were segmented into 64 × 64 pixel MSI blocks and 256 × 256 pixel PAN blocks. The dataset includes 11,000 image block pairs for training and 940 pairs for testing. Additionally, we used GF-2 satellite imagery, which was preprocessed, registered, and organized according to the Wald protocol. This dataset consists of 40,922 training image block pairs, 8769 test pairs, and 8769 validation pairs, with the multispectral images sized at 256 × 256 pixels and the corresponding panchromatic images at 1024 × 1024 pixels.

To evaluate the performance of our method, we utilized a suite of metrics including the Root Mean Square Image Error (RMSIE) [32] and Relative Average Spectral Error (RASE) [33], used to measure the performance of each band in the fused image, with an ideal value of 0.

Relative dimensionless global comprehensive error (ERGAS) [34] is defined by Equation (11); this parameter reflects the global quality of the fused image, with an ideal value of 0.

(11) $$ERGAS=\frac{100}{R}\sqrt{\frac{1}{K}\sum_{k=1}^{K}{\left(\frac{RMSE\left(I_{k},J_{k}\right)}{\mu \left(I_{k}\right)}\right)}^{2}}$$

where k represents the total number of bands, μ denotes the mean (average on pixels) of the image, and R represents the scale ratio between the source images. RMSE represents the Root Mean Square Error.

Spectral Angle Mapper (SAM) [35] is defined by Equation (12), used to calculate the absolute value of the spectral angle between vectors. Smaller values imply smaller spectral angle distortion, with an ideal value of 0.

(12) $$SAM\left(I_{\left\{i\right\}},J_{\left\{i\right\}}\right)=\arccos\left(\frac{\langle I_{\left\{i\right\},}J_{\left\{i\right\}}\rangle }{\left\|I_{\left\{i\right\}}\right\|\left\|J_{\langle i\rangle }\right\|}\right)$$

We used the Band Average Universal Image Quality Index (QAVE) [36], with an ideal value of 1.

We also employed the Spatial Correlation Coefficient (sCC), Q^2n^. The Q^2n^ is extended from the universal image quality index (UIQI). Analogously to UIQI, the Q^2n^ is defined as the product of three terms including CC, contrast distortion, and mean bias. The specific expression is as follows:

(13) $$Q^{2n}=\frac{\left|\sigma_{x\hat{x}}\right|}{\sigma_{x}\cdot \sigma_{\hat{x}}}\cdot \frac{2\sigma_{x}\cdot \sigma_{\hat{x}}}{\sigma_{x}^{2}+\sigma_{\hat{x}}^{2}}\cdot \frac{2\left|\bar{x}\right|\cdot \left|\bar{\hat{x}}\right|}{{\left|\bar{x}\right|}^{2}\cdot {\left|\bar{\hat{x}}\right|}^{2}}$$

where x = x(i, j), $\hat{x}$ = $\hat{x}$(i, j) are two hypercomplex numbers that characterize the GT and fused image at pixel (i, j), $\sigma_{x\hat{x}}$ is the covariance between x and $\hat{x}$, and $\sigma_{*}^{2}$ and $\bar{*}$ denote the variance and mean. The ideally fused image has a Q^2n^ of 1.

The no-reference quality metrics HQNR, D_s_, and D_λ_ to assess the spatial consistency and overall image quality of the fused images, ensuring a thorough evaluation of the spectral accuracy and spatial integrity of our fusion technique. Ds measures the spatial distortion complementary to Dλ, with an ideal value of 0. It is defined as Equation (14):

(14) $$D_{s}=\sqrt[{q}]{\frac{1}{C}\sum_{i=1}^{C}{\left|Q\left({\hat{x}}_{i},p\right)-Q\left(y_{i},\tilde{p}\right)\right|}^{q}}$$

where p and $\tilde{p}$ and represent PAN and its degraded LR version; q is usually set to 1.

D_λ_ is a representative spectral metric denoting the difference of interband Q values, with an ideal value of 0. Specifically, it is defined as follows:

(15) $$D_{\lambda }=\sqrt[{q}]{\frac{1}{C\left(C-1\right)}\sum_{i=1}^{C}\sum_{j=1\left(j\neq i\right)}^{C}{\left|d_{i,j}\left(\hat{x},y\right)\right|}^{q}}$$

where $\hat{x}$ and y denote the fused MS image and LR MS image, respectively. C is the number of spectral bands in the MS image, and d_i,j_ = Q($\hat{x}$_i_, $\hat{x}$_j_) − Q(y_i_, y_j_).

### 4.3. Comparative Experiments

Traditional methods including BDSD [37], BT-H [38], GS [2], and deep learning (DL) approaches such as PNN [39], TF-Net [40], FDFNet, MUCNN, DMDP, MSIDCNN, DRPNN, and DiCNN1 have been fine-tuned to their optimal parameters. These methods were compared with our proposed approach using the same training and testing samples on both the GF-2 and QuickBird (QB) datasets.

**Figure 5:**
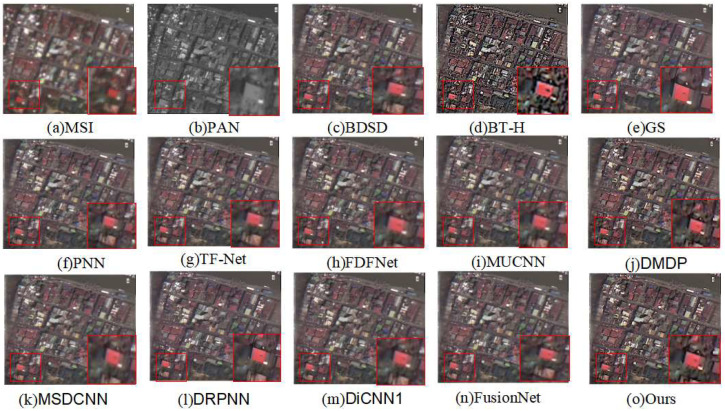
Qualitative comparison of the fused images from different methods on the QB dataset.

The fusion results on the QB dataset are illustrated in Figure 5, with the corresponding evaluation metrics listed in Table 1.

In Figure 5c–h, spectral distortion issues are observed, where the overall color reproduction is not fully achieved compared to the reference MSI. For Figure 5i–m, there is a blurring of the edges of buildings, indicating a certain loss of spatial information. The result of the method proposed in this paper is shown in Figure 5o, where the spectral information of the houses in the image is the closest to the reference image, and the edge information of the houses also shows a good effect. Therefore, from a subjective visual evaluation perspective, the method proposed in this paper has achieved a better balance in terms of spectral information fidelity and the enhancement of spatial information, resulting in a higher overall image quality.

Table 1 presents the objective evaluation metrics for the QB dataset. Objectively, the results of the objective evaluation metrics for our method outperform those of the traditional methods included in the comparison. In Table 1, Table 2, Table 3 and Table 4, the symbol ↑ indicates that the closer the data is to 1, the better the performance of the metric; the symbol ↓ indicates that the closer the value is to 0, the better the performance of the metric.

**Figure 6:**
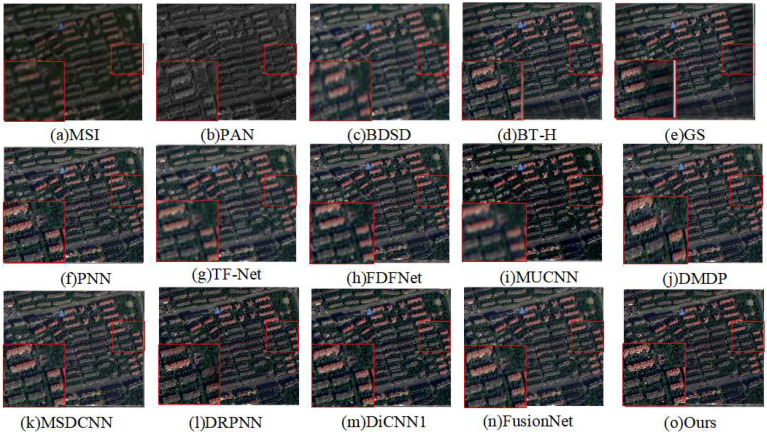
Qualitative comparison of the fused images from different methods on the GF-2 dataset.

Figure 6 presents the simulated experimental fusion results on the GF-2 satellite dataset, with the corresponding evaluation metrics listed in Table 2. From Figure 6c–e, it can be observed that the spectral information is weakened. Figure 6f shows the PNN algorithm’s result; due to the shallow depth of the PNN network, there is a blurring of building edges, and the spectral distortion issue is somewhat improved. Figure 6g,h exhibit local blurriness and information loss in the fused images. The algorithm proposed in this paper achieves better results in terms of both spectral and spatial information compared to the original MSI image, and the fused results are similar to the original MSI.

Table 2 displays the metrics from the simulated experiments on the GF-2 satellite dataset. Subjectively, our method has achieved the best results across all metrics, indicating that our approach effectively captures spectral information with minimal spectral distortion.

### 4.4. Ablation Experiments

To gain a deeper understanding of the role of the spectral–spatial embedding layer and the spatial constraint matrix in the fusion algorithm, we designed a series of ablation studies. These studies incrementally removed the spectral–spatial embedding layer and the spatial constraint matrix to assess their specific impact on the final fusion performance. In these experiments, we compared the performance of the original algorithm (which includes the spatial constraint matrix) with the ablated versions (which do not include the spectral–spatial embedding layer or the spatial constraint matrix).

Table 3 presents the quantitative results of the ablation experiments on the same dataset. Through comparative analysis, we found that when the spectral–spatial embedding layer was removed and only the traditional embedding layer of Mamba was used, the final fusion results exhibited a loss of spectral information. Additionally, after the removal of the spatial constraint matrix, the algorithm experienced a decline in performance across six objective metrics, including RASE. This reveals the importance of the spatial constraint matrix when dealing with complex terrain and geographic features.

## 5. Discussion

In this section, our method is compared with five of the latest studies in the field of remote sensing image fusion: Paper 1 [41], Paper 2 [42], Paper 3 [43], Paper 4 [44], and Paper 5 [45]. These studies were selected for their relevance to our research and their use of similar datasets and evaluation metrics.

Paper 1 proposed a deep learning-based fusion network that enhances the fusion quality of remote sensing images through multi-scale feature extraction. Unlike this approach, our method introduces a spectral–spatial embedding layer and a spatial constraint matrix, significantly enhancing the model’s ability to process three-dimensional remote sensing data. This combination allows our model to better preserve spectral information and spatial details when handling complex terrain and feature characteristics. Paper 2 utilized a U-Net based architecture, employing an attention mechanism to enhance the edges and textures of the fused image. In contrast, our method incorporates a Spatial Proximity Perception Attention Module (SPPAM), an advanced attention mechanism that improves the capture of complex geographical object relationships by dynamically adjusting the weights between feature maps. Paper 3 presented a Transformer-based model that leverages self-attention mechanisms to capture long-range dependencies. Our approach achieves a better balance between spectral and spatial information, introducing a Spatial Proximity Constraint Gated Fusion Module (SPCRGF) that reduces spectral distortion and spatial structure loss. Paper 4 employed a multi-modal fusion network that improves fusion performance by jointly optimizing spectral and spatial information. Our method significantly enhances the capture and emphasis of key geographical feature details through precise spatial constraints, particularly excelling in practical applications such as power grid surveys. Paper 5 proposed a fusion network based on multi-spectral feature enhancement, using multi-frequency analysis to improve the quality of the fused image. Our method has the advantage of reducing spectral distortion and spatial structure loss, achieving a higher QAVE and demonstrating better overall image quality. See Table 4.

The table indicates that our method outperforms Papers 1, 2, 3, 4, and 5 in terms of RMSIE, RASE, ERGAS, and SAM, demonstrating lower spectral distortion. Furthermore, our method achieves a higher QAVE, showcasing superior overall image quality.

The superiority of our method can be attributed to the introduction of the Spatial Proximity Constraint Gated Fusion Module (SPCRGF), which significantly improves the capture and emphasis of key geographical feature details through precise spatial constraints. This advantage is particularly evident in practical applications such as power grid surveys, where it is necessary to highlight the terrain and geographical features surrounding power lines during the fusion process.

However, our method also has some limitations. For instance, the computational complexity of our model is relatively high, which may affect its efficiency in real-time applications. Future work will focus on introducing cross-modality attention mechanisms to enhance the intrinsic connections between PAN image and MSI channels and reduce the model’s computational complexity.

In summary, compared to existing methods, our approach demonstrates significant improvements in spectral accuracy and spatial integrity. The introduction of advanced attention mechanisms and spatial constraints makes our method particularly effective in practical applications such as power grid surveys.

## 6. Conclusions

In this article, we successfully proposed an innovative multi-scale progressive gating fusion model, which is based on Mamba and attention mechanisms, effectively addressing the challenges of panchromatic and hyperspectral image fusion in the field of power grids. By introducing a spectral–spatial embedding layer, we effectively integrated spatial and spectral features, significantly enhancing the model’s ability to process three-dimensional remote sensing data. Furthermore, the TransforRS-Mamba model, which combines the advantages of sequence processing and attention mechanisms, enhances the capture of complex geographic object relationships through the Spatial Proximity Perception Attention Module (SPPAM). Most critically, the introduction of the Spatial Proximity Constraint Gating Fusion Module (SPCRGF) significantly improves the capture and emphasis of key geographic feature details through precise spatial constraints, demonstrating outstanding performance in practical applications such as power grid surveying.

The experimental results indicate that our method can effectively balance spectral and spatial structural information, reducing spectral distortion and the loss of spatial structure. Compared to the comparative algorithms, the model we adopted is more significant in the application of power grid surveying, highlighting the surrounding terrain and geographic features of the power lines during the fusion process, assisting designers in avoiding buildings, vegetation, and rivers.

While this study has achieved positive results, there are some limitations to acknowledge: First, the model has primarily been tested on power grid survey data, and its generalizability to other types of remote sensing data or different geographical regions remains unverified. Despite these limitations, the outcomes of this study hold practical application potential in several fields, such as power grid surveying, environmental monitoring, and urban planning. To overcome the current limitations and further enhance model performance, future research will focus on incorporating cross-modality attention mechanisms, optimizing the model to reduce computational complexity, and improving the model’s robustness to parameter selection. With these improvements, we anticipate advancing remote sensing technology and expanding its impact in a broader range of practical applications.

## Figures and Tables

**Table 1:** Quantitative representation in the QB dataset.

Method	RMSIE↓	RASE↓	ERGAS↓	SAM↓	QAVE↑
BDSD	3.89	16.52	4.85	2.5715	0.9561
BT-H	3.15	12.84	4.42	2.2841	0.9584
GS	2.64	15.38	3.29	2.3642	0.9263
PNN	2.98	13.59	2.65	1.4786	0.9452
TF-Net	1.42	9.43	1.84	2.1572	0.9388
FDFNet	1.78	6.15	2.17	1.7688	0.9815
MUCNN	1.26	8.64	2.09	2.6909	0.9462
DMDP	2.51	7.58	2.45	2.5462	0.9041
MSIDCNN	3.45	6.11	1.99	1.6840	0.8543
DRPNN	2.91	7.46	3.68	2.0064	0.9345
DiCNN1	1.46	6.09	2.93	1.5647	0.9480
Ours	1.10	5.94	1.52	1.3469	0.9941

**Table 2:** Quantitative representation in the GF-2 dataset.

Method	RMSIE↓	RASE↓	ERGAS↓	SAM↓	QAVE↑
BDSD	8.56	21.52	3.51	6.9640	0.5015
BT-H	7.29	20.38	2.94	7.1164	0.3749
GS	6.34	16.73	3.16	6.8129	0.7423
PNN	7.16	19.84	3.09	5.3545	0.8634
TF-Net	5.49	16.33	2.84	3.9427	0.8427
FDFNet	4.63	13.26	2.69	2.1254	0.7931
MUCNN	4.68	15.98	2.73	2.6738	0.7165
DMDP	6.54	16.75	3.55	2.4896	0.3548
MSIDCNN	4.58	13.84	2.84	3.5489	0.4541
DRPNN	7.12	15.41	3.25	3.4831	0.3484
DiCNN1	4.67	16.07	2.94	2.6844	0.7860
Ours	3.51	13.19	2.58	2.0540	0.8947

**Table 3:** Ablation experiments.

Method	RASE↓	ERGAS↓	SAM↓	Q^2n^↑	D_s_↓	D_λ_↓
Remove the empty spectral embedding layer	6.92 ± 0.12	2.53 ± 0.04	3.02 ± 0.11	0.548	0.3454	0.055
Remove the spatial constraint matrix	8.14 ± 0.21	1.95 ± 0.08	2.88 ± 0.03	0.863	0.1843	0.041
Ours	5.37 ± 0.03	1.64 ± 0.02	1.05 ± 0.21	0.942	0.0042	0.0027

**Table 4:** Quantitative comparison of our method with Paper 1, Paper 2, Paper 3, Paper 4, and Paper 5 on the QB dataset.

Method	RMSIE↓	RASE↓	ERGAS↓	SAM↓	QAVE↑
Paper 1	3.54	16.84	4.75	8.73	0.3215
Paper 2	4.26	15.49	4.31	7.60	0.5644
Paper 3	3.48	16.02	5.26	8.35	0.1486
Paper 4	4.07	14.73	3.49	7.49	0.3647
Paper 5	4.60	15.35	5.07	6.91	0.6447
Ours	3.14	12.94	3.16	5.36	0.8391

## Data Availability

Data are contained within the article.

## Abbreviations

SPPAM	Spatial proximity perceives attention mechanism
MSI	Multispectral image
PAN	Panchromatic
CS	Component substitution
MRA	Multiresolution analysis
VO	Variational optimization
SSM	State Space Model
ZOH	Zero-order hold
PCA	Principal Component Analysis
ROSSM	Residual Omnidirectional Selective Scan Module
Ed	Euclidean distance
Dd	Dot-product distance
Md	Mahalanobis Distance
RMSIE	Root Mean Square Image Error
RASE	Relative Average Spectral Error
SAM	Spectral Angle Mapper
ERGAS	Relative dimensionless global comprehensive error
UIQI	Universal Image Quality Index
PSNR	Peak Signal-to-Noise Ratio
SPCGF	Spatial Proximity Constraint Gated Fusion Module
